# Deep Tubewell Use and Child Diarrhea in Rural Bangladesh: Results from a Prospective Community Surveillance Study

**DOI:** 10.1021/EHP.6c00045

**Published:** 2026-04-22

**Authors:** Varun Goel, Mia Ziade, Brianna Chan, Md. Yunus, Md. Taslim Ali, Md. Al Fazal Khan, Md. Nurul Alam, ASG Faruque, Shahabuddin Babu, Md. Masnoon Kabir, Paul L. Delamater, Marc L. Serre, Mark D. Sobsey, Md. Sirajul Islam, Michael Emch

**Affiliations:** † Department of Geography, University of South Carolina, Columbia, South Carolina 29208, United States; ‡ Carolina Population Center, University of North Carolina-Chapel Hill, Chapel Hill, North Carolina 27599, United States; § Department of Statistics, University of North Carolina-Chapel Hill, Chapel Hill, North Carolina 27599, United States; ∥ Gillings School of Global Public Health, University of North Carolina-Chapel Hill, Chapel Hill, North Carolina 27599, United States; ⊥ International Centre for Diarrhoeal Disease Research, Bangladesh (icddr,b), Dhaka 1212, Bangladesh; # Department of Geography, University of North Carolina-Chapel Hill, Chapel Hill, North Carolina 27599, United States

## Abstract

**BACKGROUND:** Diarrheal diseases remain a leading cause of mortality and morbidity among under-five children in South Asia. In rural Bangladesh, deep tubewells that tap into low-arsenic deep aquifers have been installed to provide microbially safe and arsenic-free drinking water at the source. However, unlike more widely used shallow tubewells, deep tubewells are sparsely distributed, and households often travel farther for drinking-water consumption from such wells. Hence, benefits from deep tubewells may be abated by higher levels of microbial contamination during water handling and storage that could increase the risk of diarrheal diseases. **OBJECTIVES:** We examined the association between deep tubewell use and diarrheal disease risk in under-five children and investigated the role of social and environmental factors on modifying the association. **METHODS:** We implemented community diarrheal disease surveillance across households with under-five children using deep and shallow tubewells in Matlab, Bangladesh from March 2018 to October 2019. We used Generalized Estimating Equations (GEE) to estimate the association between deep tubewell versus shallow tubewell use and diarrheal disease prevalence. **RESULTS:** Children in households using deep tubewells had diarrheal disease prevalence 0.83 times that of children in households using shallow tubewells (95% confidence interval (CI): 0.71–0.96). Protective effects of deep tubewell use on diarrhea risk were observed among children in households that drank from wells within their household compound (risk ratio (RR) = 0.70, 95% CI: 0.54 – 0.91), were in flood-prone areas (RR = 0.83, 95% CI: 0.75 – 0.92), and used unimproved latrines (RR = 0.62, 95% CI: 0.43–0.89). Deep tubewell use was more protective against diarrhea than shallow tubewell use during the dry season (RR = 0.71, 95% CI: 0.52–0.97). **CONCLUSIONS:** Despite concerns, using deep tubewells may not translate to higher diarrhea risk among under-five children and may reduce diarrhea further, especially in social and environmental contexts associated with higher groundwater microbial contamination.

## 1 Introduction

Despite substantial public health advances, diarrheal diseases remain a leading cause of mortality and morbidity among children under five years of age,^1^ with 90% of attributable deaths occurring in South Asia and Sub-Saharan Africa.^2^ In Bangladesh, the diarrheal disease burden among children under five has substantially declined, and this reduction is partially attributed to a universal shift among rural Bangladeshis from drinking surface water to groundwater, facilitated by the installation of millions of accessible and inexpensive shallow tubewells that tap into the shallow aquifer less than 140 feet.^1–4^

However, the discovery of widespread naturally occurring arsenic in groundwater at shallow depths across Bangladesh exposed millions of residents to a new public health emergency linked to arsenic poisoning.^4^ In response, households switched drinking from their high-arsenic shallow tubewells that were deemed unsafe to neighboring low-arsenic tubewells that were deemed safe based on widespread arsenic testing.^5,6^ Groundwater pumped from shallow low-arsenic wells, however, may be more contaminated with human feces than groundwater from shallow high-arsenic wells due to the local hydrogeological factors linked to rapid downward transport and less efficient filtration of microbial contaminants, high population density, and poor sanitation.^7–11^ Hence, avoiding arsenic contamination by switching to low-arsenic shallow tubewells may inadvertently be linked to higher diarrheal disease risk,^12^ and fecal contamination of shallow groundwater may be a factor in the persistence of diarrheal diseases in rural Bangladesh.^8,13,14^

Another significant arsenic mitigation strategy followed by one out of every four rural households involves sourcing drinking-water from deep tubewells with depths exceeding 500 feet.^15^ These deep tubewells tap into older geological strata free of arsenic and are generally free of fecal contamination due to increased depths and physical separation from transport of land surface and shallow subsurface fecal materials, contributing to safer drinking-water quality at source.^16–20^ However, in practice, deep tubewell use may also contribute to the consumption of unsafe drinking-water due to barriers to availability and access. Deep tubewells routinely cost five to ten times more than shallow tubewells, require more technical expertise for construction,^21^ and are often sparsely and inequitably located across rural Bangladesh.^22^ As a result, fewer households can afford to own a deep tubewell and often rely on farther deep tubewells owned by other nearby households or public deep tubewells installed in community areas such as a school, religious building (e.g., mosque), or a market.

Hence, lower exposure to microbial contamination at source may be offset by additional risks of recontamination during transport, water handling and storage.^23^ Compared to households using shallow tubewells for drinking water, fewer households own deep tubewells, and those using deep tubewells walk farther to their drinking-water source, store water longer, and use microbially unsafe surface water sources more frequently for other nonconsumption tasks.^24^ Subsequently, deep tubewell use may be associated with higher microbial contamination at point-of-use (POU)^25^ and may instead be linked to higher risk of diarrheal diseases among rural Bangladeshis in certain contexts.^14,26^

It is unclear, however, whether deep tubewell use is associated with diarrheal disease risk. Earlier evidence suggests that while deep tubewell use is linked to lower diarrheal disease risk in one part, it may be associated with higher mortality in another part of rural Bangladesh.^27–29^ However, there are two major gaps associated with the role of deep tubewells in diarrheal disease risk in rural Bangladesh. First, the factors that may drive these contrasting associations have not been systematically investigated, thus limiting our understanding of the effectiveness of deep tubewells in providing safe drinking water and health benefits across various settings in rural Bangladesh. It is possible that the relationship between deep tubewell use and diarrheal disease risk is context-dependent and that there may be important social and environmental factors that determine whether deep tubewells protect against or exacerbate diarrheal disease risk among children under five years of age. Second, all previous studies investigating deep tubewell use and diarrhea were either limited in spatial scope,^28^ or made strong assumptions about household tubewell use. For example, in most longitudinal studies it was assumed that households either used the same tubewell as their primary drinking-water source across time, or households were assumed to be either using a shallow or deep tubewell based on proximity to the nearest tubewell source.^13,27,29^ However, these assumptions may fail to take into account other important preferences in choosing a safe drinking-water source^30–32^ or may not be realistic in the current context of drinking-water provision in rural Bangladesh where households may have multiple options and variability in drinking-water use and security over time.^33,34^

Our study addresses these empirical gaps by implementing a longitudinal community level diarrhea surveillance study that can be linked to detailed demographic, social, and environmental data across a diverse geographic area. The primary objective of this paper is to examine differences in the risk of diarrheal disease among children in households using deep tubewells or shallow tubewells and examine factors associated with those differences. We examine diarrheal disease prevalence among children under five years of age in households using deep or shallow tubewells and its association with tubewell type using comprehensive, population-based active community surveillance. We then further examine the social and environmental factors that may modify the relationship between deep tubewell use and diarrheal disease risk in under-five children.

## 2 Data and Methods

### 2.1 Study Area

We conducted the study in Matlab, Bangladesh, a rural region spanning 184 km^2^ and located 55 km southeast of Dhaka within the Chandpur district in the Chattogram Division (formerly Chittagong Division). Matlab has 142 villages, and as of 2018, a total population of approximately 240,000 individuals and 59,000 households.^35^ It is a field research site for the International Centre for Diarrheal Disease Research, Bangladesh (icddr,b), which has had an extensive Health and Demographic Surveillance System (HDSS) in place since 1966, which maintains vital demographic records from each household in the area. As part of the HDSS data collection, community health research workers (CHRWs) routinely visit each household once every 2–3 months and collect information about events such as births, deaths, and migration. Households in Matlab, like other parts of rural Bangladesh are arranged spatially based on patrilineal linkages in clusters called *baris*. A *bari* contains up to a dozen households, with an average of five to six households per *bari*. All members of a *bari* community generally drink from a shared tubewell source. Those *baris* that do not own a tubewell obtain drinking-water from water sources in neighboring *baris* or other communities or facilities, such as a school, a religious building, or a public marketplace. Due to the regular occurrence of flooding in the study area, a large flood control embankment was completed in 1990 that roughly divided the physical area of the HDSS in half (Figure 1).

**Figure 1:**
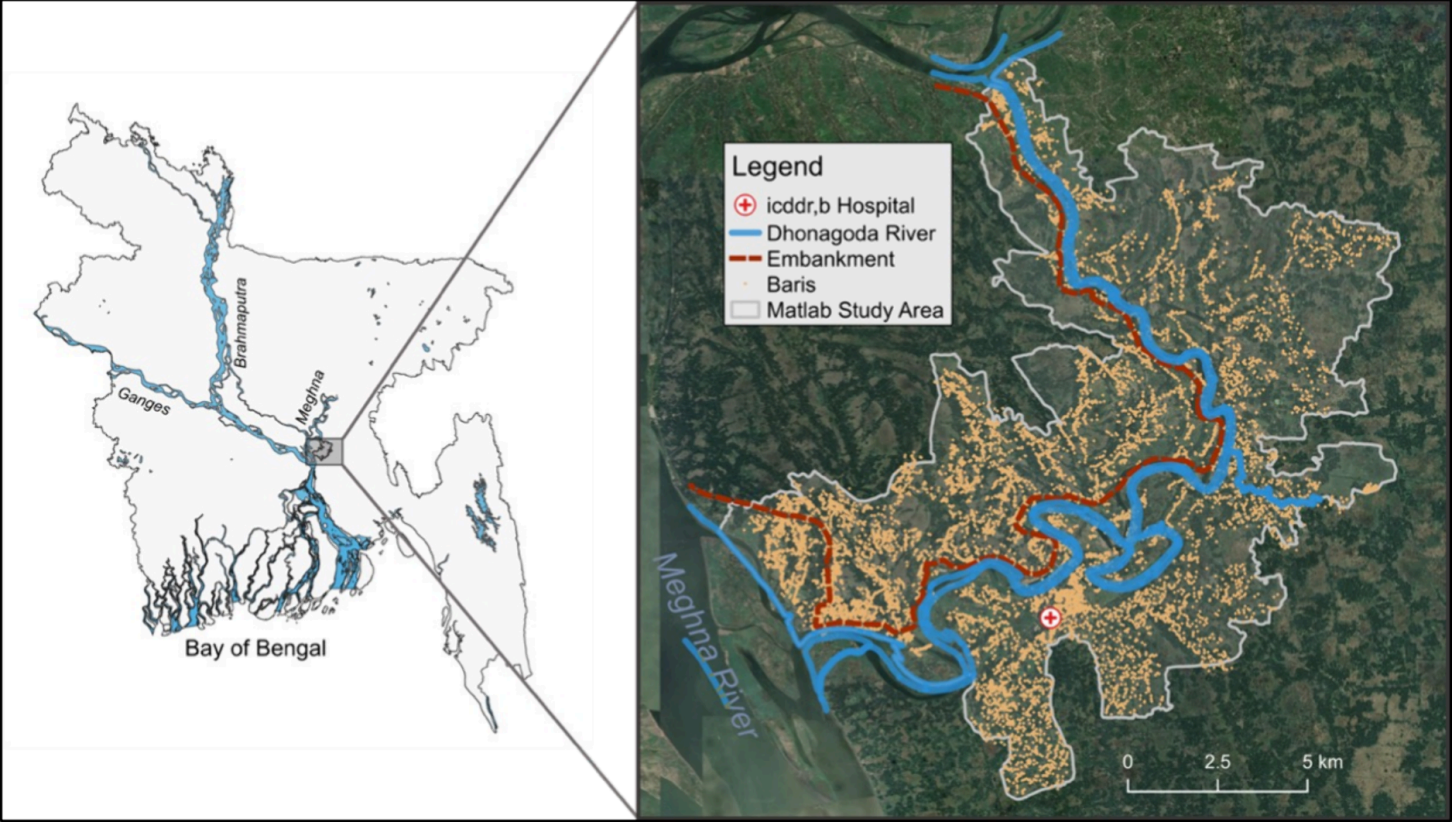
Study area map of Matlab, Bangladesh. *Bari* locations are based on shapefiles provided by icddr,b as part of the 2014 Household Socioeconomic Census in Matlab. *Baris* to the west of the embankment are within the flood control zone.

Based on a blanket arsenic testing campaign in 2002–03, Matlab had pervasive groundwater arsenic contamination with two out of every three wells containing arsenic concentrations greater than the Bangladeshi permissible limit of 50 μg/L.^21^ Despite widespread deep tubewell installations and interventions since then,^36^ high arsenic exposure and diarrheal disease burden remain a challenge. As of 2015, only 15% households reported using deep tubewells and 27% of households procured their drinking-water from untested or arsenic-unsafe tubewell sources.^37^ Although updated diarrheal disease estimates were not available for Matlab since 2006, results for a country-wide population-based survey reported an 8% diarrhea prevalence among children under five in Chandpur district.^38^

### 2.2 Study Design and Participants

As part of our study, we conducted community surveillance for diarrheal disease events from March 2018 to October 2019 to assess the risk of diarrheal diseases among households that had at least one under-five child and used either a deep or shallow tubewell as their primary source of drinking-water. Another purpose of the community surveillance system was to update under-five diarrheal disease estimates in Matlab and link them to tubewell use, as no population-level under-five diarrheal disease data collection had taken place in Matlab since 2006. On their routine visits (approximately once every three-four months), CHRWs determined whether a household had at least one child younger than five years, and if so, they asked the mother (or household head in case the mother was not present) about whether the household obtained their drinking-water from a shallow or deep tubewell. If both inclusion criteria were satisfied, the CHRWs recorded whether the household members obtained drinking-water from a well in their own *bari*, another *bari*, or if they used a community well. The type of tubewell used was based on self-reported information by a household member. While there may be potential misclassification bias in the type of tubewell used due to self-reporting, tubewell owners were also asked about tubewell depth as they are aware of tubewell depth based on the polyvinyl chloride (PVC) pipe length needed for tubewell installation. Additionally, most households in Matlab are sensitized to the difference between deep and shallow tubewells due to numerous deep tubewell installations and arsenic mitigation campaigns over the past two decades. In this study, tubewells with depths greater than 150 m were classified as deep tubewells, consistent with deep tubewell classifications in previous studies,^5,27,28,39^ and by the Department of Public Health Engineering (DPHE) which is responsible for deep tubewell installations across Bangladesh.

CHRWs recorded the diarrhea status of each child under five years of age, based on the World Health Organization (WHO) definition of three or more watery stools within 24 h in the preceding 2 days. Additionally, diarrheal disease episodes that were reported to continue for several days were recorded as a single case. The primary outcome of interest was the prevalence proportion of households that had at least one under-five child with reported diarrhea over the 2 days prior to a CHRW visit. CHRWs used standard informed consent forms for explaining the study purpose and guaranteeing the confidentiality of information. Participant willingness was expressed by either signature or thumb impression. Ethical approval for this study was obtained from the University of North Carolina Institutional Review Board and the icddr,b Ethical Review Committee.

### 2.3 Household Socioeconomic Census

The icddr,b conducted a household socioeconomic census (HSEC) of the *de jure* population in 2014.^37^ Individual-level and structured household-level questionnaires were administered to collect information about key household-level social and economic characteristics. Some key characteristics included data on mother’s education, occupation of household head, household income, assets and commodities, sources of drinking-water and sanitation, access to services including support from nongovernmental organizations and government service information centers (called Union Information Centers). Similar to the methodology used in Demographic and Household Surveys (DHS),^40^ a wealth index in the form of wealth quintiles was computed for each household. The 2014 HSEC also recorded whether households used a shallow or deep tubewell along with information on the arsenic testing status of the shallow tubewell. Out of all households recorded in our community surveillance, 80% were matched to household level data from the 2014 HSEC based on unique household identifiers that are assigned to each household within the Matlab HDSS. These unique household identifiers enable longitudinal analysis and data linkage across multiple demographic and health data sources.^35^ Of the remaining households that did not match to household level baseline characteristics from the 2014 HSEC, 85% were assigned SES characteristics based on a matching *bari*, while the remaining households were assigned baseline characteristics based on a matching village. *Bari*- and village-level baseline characteristics were computed by taking the mean or sum of each household-level covariate for all households in the same *bari* or village from the 2014 HSEC data. *Bari*-level indicators such as SES scores and mother’s education have been constructed and utilized as covariates in previous studies.^27,41^

### 2.4 Statistical Analysis

We examined the distribution of baseline characteristics among deep and shallow tubewell users based on the reported tubewell type by a household during community surveillance. We included baseline characteristics that were relevant to the context of both tubewell choice and diarrheal diseases in rural Bangladesh. Tubewell ownership, characterized by whether a household used a tubewell inside their own *bari*, someone else’s *bari*, or a community well, is expected to differ between deep and shallow tubewell users due to higher financial costs associated with deep tubewell ownership. Additionally, it is also correlated with distance to drinking-water source,^33^ which subsequently has been linked to higher risk of diarrheal diseases.^42^ In addition to confounders such as household wealth and level of mother’s education, we also include characteristics about household latrine usage and type, household drinking-water treatment, access to economic and knowledge institutions, and whether a household was located within the flood control embankment or outside of it.

While household latrine use may not be directly linked with the type of tubewell used, it may be an important effect modifier and influence the relationship between tubewell type and diarrhea due to the potential for source contamination of groundwater.^43,44^ Membership in NGOs has been shown to be associated with more knowledge about unsafe water sources, and the availability of resources to pay for the installation of arsenic safe water sources such as deep tubewells.^45^ Similarly, Union Information Centers often provide valuable health information to households in remote areas,^46^ and may potentially influence diarrheal disease risk and use of safe drinking-water sources. We linked households in the community surveillance to the household rosters in the HDSS to determine whether households were inside or outside of the flood control embankment. Previous studies have shown that being inside or outside the flood control embankment is linked to differences in tubewell density,^13^ diarrheal disease risk, type, and seasonality.^47–50^

We summarized the distribution of diarrhea prevalence for both tubewell groups across the study period, followed by temporal trends of recorded diarrhea cases across the study period and seasons. Matlab has a tropical-monsoon climate and typically experiences three seasons: a cool and dry season (November – February), a hot summer season (March – June), and a peak rainy season (July – October).^51^ Several studies evaluating long-term trends in diarrheal disease burden have shown significant seasonal variations.^52–54^ Additionally, seasonality may also be linked to tubewell switching and variations in tubewell use. While most households in Matlab may have access to multiple tubewells, both the dry and rainy seasons may influence households’ primary drinking-water source preferences. During the rainy season, heavy flooding, especially in areas outside the flood control embankment, can make it difficult for households to access their preferred drinking-water source. Similarly, the prolonged dry season may cause some shallow tubewells to become dry, prompting households to travel to alternative tubewells with available drinking-water.^34^

We estimated the association between deep tubewell use and diarrheal disease risk using Generalized Estimating Equations (GEEs) with a binomial log link function under the assumption of an exchangeable correlation matrix and robust sandwich estimators to account for repeated household visits.^55^ An exchangeable correlation matrix assumes that observations for the same household are equally correlated and not dependent on the time at which they were measured. In contrast to an autoregressive correlation structure that assumes that observations closer to each other in time are more correlated, we chose an exchangeable correlation matrix due to the unbalanced nature of our observations across the study period and to account for the fact that past diarrhea episodes may not protect children from future diarrheal disease risk. We included covariates in our model based on a conceptual directed acyclic graph (DAG) (Supplemental Figure 1) to account for measured confounding based on the theoretical expectation of relationships between our covariates and diarrheal disease risk. The minimum set of adjustment covariates include household wealth, mother’s education, status of tubewell ownership, and seasonality. We also controlled for the total number of under-five children in the household during the visit and for the effect of any missing covariate data by adding indicator variables for *bari* and village level imputation. Households that could not be matched to the 2014 socioeconomic census were assigned mean baseline socioeconomic characteristics for all other households within a *bari*, followed by the village.

We then assessed whether the association between deep tubewell use and diarrheal disease risk was modified by household-level factors such as ownership, seasonality, location within the flood control embankment, wealth and mother’s education, and access to economic and knowledge institutions such as NGOs or Union Information Centers. We measured effect measure modification at the ratio scale, and assessed model fit for added interaction terms using the Quasi Information Criterion (QIC).^56^ We displayed stratum-specific estimates of deep tubewell use on the diarrhea risk for all potential effect modifiers in Figure 3. DAGs were created using Daggity v3.0,^57^ and statistical analysis was conducted in R v4.0.^58^ We conducted GEE analysis using the ‘geepack’ package,^59^ assessed model fit using the ‘MuMIn’ package,^60^ and estimated within and between stratum comparisons using the ‘contrast’ package.^61^

## 3 Results

### 3.1 Household Baseline Characteristics

During the diarrheal community surveillance period, CHRWs made over 100,000 visits to households satisfying the inclusion criteria, and a respondent was present 85% of the time. After removing duplicates and nonresponses, a total of 84,425 visits were recorded in the final data set. Out of these, households reported using a deep tubewell 18% of the time. In total, 22,744 unique households were visited, with a median of three visits per household (IQR: 2–5). Out of these unique households, 79% used a shallow tubewell as their primary drinking-water source throughout the study period compared to 13% that used only a deep tubewell. 7.3% of households reported using both a deep and a shallow tubewell at different visits during the study period.

Households that reported using a deep tubewell as compared to shallow tubewell primarily differed based on the ownership of the tubewell from where they procured their drinking-water at the time of a CHRW visit (Table 1). Households that obtained drinking-water from deep tubewells reported using a deep tubewell inside their *bari* 44% of the time, followed by the deep tubewells in another *bari* (34%), and the use of community deep tubewells (22%). In contrast, households using shallow tubewells reported using a shallow tubewell inside their *bari* 87% of the time, followed by using a tubewell outside their *bari* 11% of the time. There were no significant differences in other social, economic, or environmental characteristics among households using shallow or deep tubewells.

**Table 1:** Baseline Characteristics of Households in Prospective Community Surveillance Study That Use Shallow and Deep Tubewells As Their Primary Drinking-Water Source^a^

	Deep Tubewell (%)	Shallow Tubewell (%)
*Tubewell Ownership* ^a^		
inside *bari*	2566 (43.76)	18675 (86.61)
outside *bari*	1994 (34.00)	2438 (11.31)
community	1304 (22.24)	448 (2.08)
*Use Based on Seasonality*		
dry season	2395 (26)	10845 (27)
hot/summer season	3573 (38)	15902 (38)
rainy season	3309 (35)	14411 (35)
*Wealth Index* ^b^		
1 (poorest)	754 (16.15)	3158 (18.57)
2	985 (21.09)	3358 (19.75)
3	986 (21.11)	3504 (20.61)
4	1088 (23.30)	3755 (22.09)
5 (richest)	857 (18.35)	3227 (18.98)
missing	0	14
*Mother’s Education*		
median years of education (IQR)^c^	3 (0–6)	3 (0–6)
*Filtration Method*		
use of any type of filter for drinking	519 (11.11)	1820 (10.70)
no use of any type of filter for drinking	4151 (88.89)	15196 (89.30)
*Latrine Use*		
latrine ownership	4249 (90.99)	15513 (91.17)
no latrine ownership	421 (9.01)	1503 (8.83)
*Latrine Type and Sharing*		
improved (not shared)	2541 (54.40)	9705 (57.03)
improved (shared)	1426 (30.53)	4877 (28.66)
unimproved	704 (15.07)	2434 (14.30)
No. of households sharing latrine (IQR)	0 (0.2)	0 (0.2)
*Access to Institutions*		
NGO membership	1871 (40.06)	6877 (40.41)
no NGO membership	2799 (59.94)	10139 (59.59)
knowledge about Union Information Center	1292 (27.67)	3899 (22.91)
no knowledge of center	3378 (72.33)	13117 (77.09)
visit to Union Information Center	624 (13.36)	1479 (8.69)
no visit to Union Information Center	4046 (86.64)	15537 (91.31)
*Embankment*		
household inside embankment	1493 (32.27)	5509 (32.75)
household outside embankment	3134 (67.73)	11313 (67.25)
missing	43	194
*Number of Children*		
mean children under five years of age ± SD	1.19 ± 0.46	1.20 ± 0.45

a All baseline characteristics other than tubewell ownership were computed from households matched to the 2014 Household Socioeconomic Census (17919 out of 22744 households). Households reporting more than one tubewell use or ownership are repeated more than once in respective categories. All values are reported as *n* (% of households) unless otherwise noted.

b Tubewell ownership and use based on seasonality characteristics were obtained from the community surveillance survey.

c Wealth index and all other variables below were obtained from the 2014 Matlab Health and Socioeconomic Survey.

d IQR – Interquartile range.

### 3.2 Diarrheal Disease Outcomes

Across all CHRW visits, 287 under-five child diarrhea cases out of 15,596 CHRW visits (1.84%) were recorded in households procuring their drinking-water from deep tubewells, while 1462 cases out of 69,873 visits (2.09%) were recorded among households that used shallow tubewells. Additionally, among the households that reported using deep tubewells, 264 out of 4685 (5.6%) households reported at least one case of diarrhea among children under five years of age. Similarly, 1299 out of 19729 (6.6%) households that reported using shallow tubewells also reported at least one case of diarrhea among children under five years of age during the community surveillance period.

The number of CHRW visits was lowest in the months of October through February since these months were only included once during the study period (Figure 2A). Hence the sample size of visits, especially for deep tubewells was lower during the dry season. Trends of diarrheal disease cases by month suggest mostly similar patterns among shallow and deep tubewell users (Figure 2B). According to Figure 2C, a large peak in diarrheal disease prevalence is observed in May (i.e., the end of the hot/summer season) among both deep and shallow tubewell households, followed by a smaller peak in October (i.e., the end of the rainy season) among deep tubewell users and November to December (i.e., the dry season) among shallow tubewell users.

**Figure 2:**
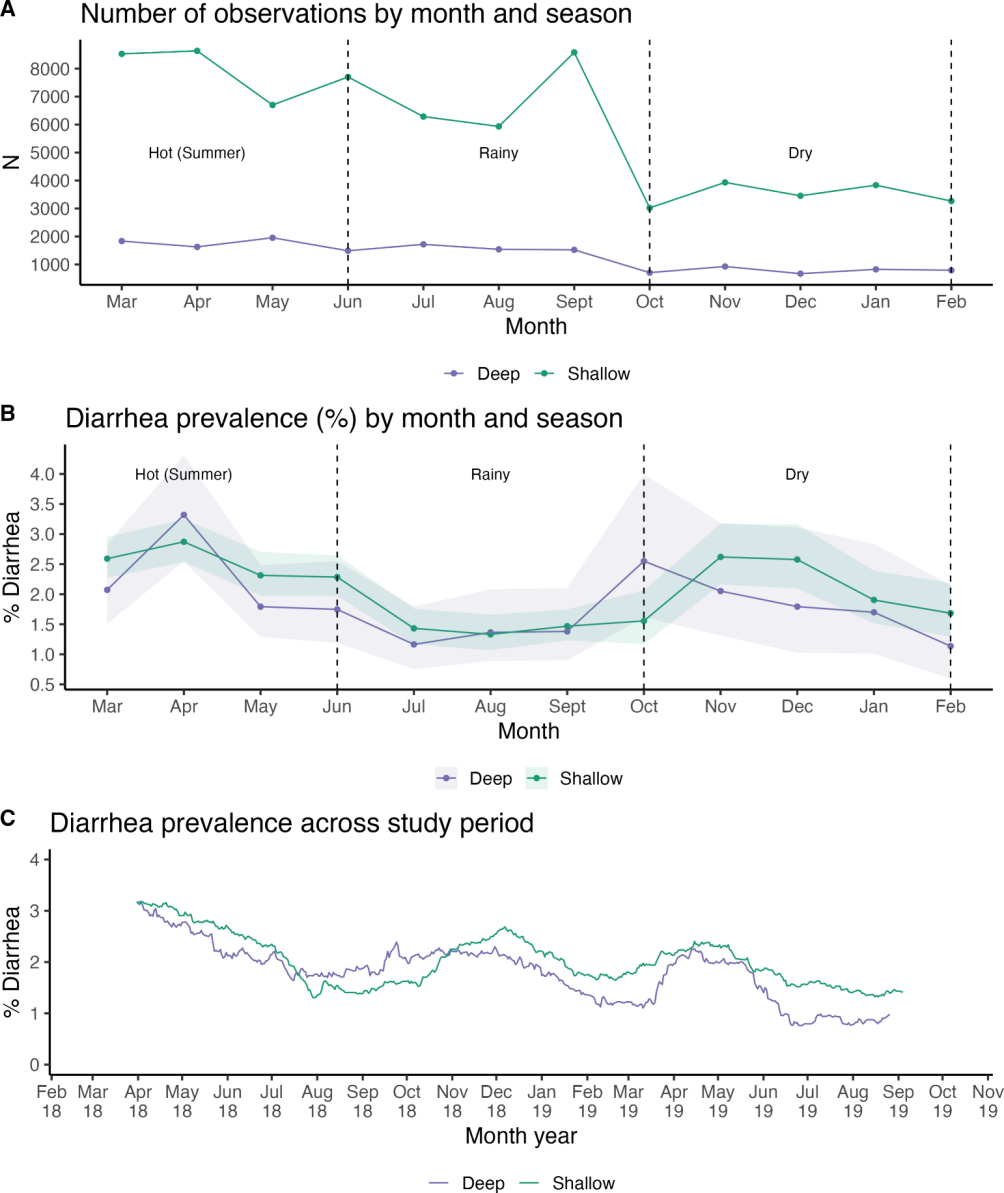
Temporal trends of diarrhea prevalence across study period. (A) Distribution of total household responses by month and season. (B) Diarrhea prevalence (number of households with diarrhea cases/number of responses) by month and season. (C) Mean-centered two month rolling average of diarrhea prevalence across the study period ranging from March 1, 2018 to October 6, 2019. Note: Estimates for April 18 and Sept 19 are missing due to using a mean centered rolling average. Numerical estimates are available in Excel Tables S1, S2, and S3.

### 3.3 Model Results

According to the adjusted model results, households using deep tubewells were 0.83 (95% CI: 0.71–0.96) times as likely to have a child under five years of age with diarrhea as compared to households using shallow tubewells, controlling for all other factors and accounting for multiple household observations across time (Table 2). Model fit based on a quasi-information criterion (QIC) suggested that there was no significant effect modification on the ratio scale between strata for any of the effect modification variables. However, significant effect sizes associated with deep tubewell use and diarrheal disease risk were observed within specific strata for some variables (Figure 3). Among households procuring drinking-water from a tubewell in another *bari*, deep tubewell users had 0.70 (95% CI: 0.54–0.91) times the risk of diarrheal disease compared to shallow tubewell users (Figure 3A). Although point estimates of relative risk were protective among both households obtaining drinking-water in their own *bari* and in the community, the confidence intervals also included the potential for harm. On comparing users across ownership strata, we found that deep tubewell users drinking from a community well or a well in another *bari*, compared to shallow tubewell users drinking from a well within their own *bari*, were not at higher risk for diarrheal disease. Additionally, diarrheal disease risk was not significantly higher in households that use a deep tubewell in the community or another *bari* compared to deep tubewell users drinking water from a well in their own *bari*.

**Figure 3:**
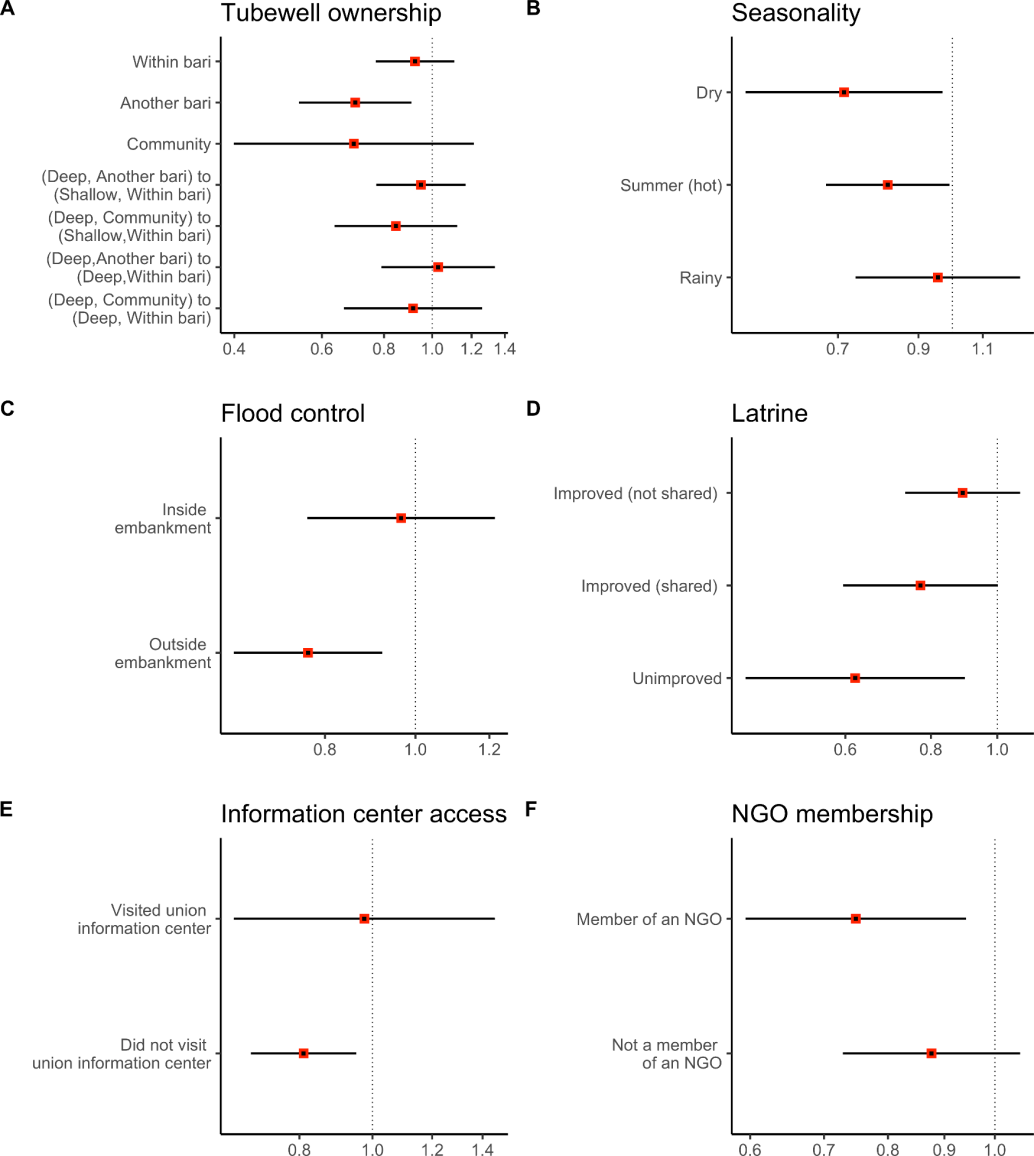
Stratum-specific estimates of tubewell use on diarrheal disease across main effect modifiers. Forest plots show stratum-specific adjusted risk ratios (95% CI) of deep tubewell use, compared to shallow tubewell use on diarrheal disease for main effect modifiers. Numerical estimates for all modifiers are available in Excel Table S5.

**Table 2:** Model Results of Analysis Comparing Risk Factor Associations with Under-five Diarrhea Prevalence^a^

Variables	Crude RR (95% CI)	Adjusted RR (95% CI)
*Tubewell Use*		
shallow tubewell	REF	
deep tubewell	0.89 (0.77–1.01)	0.83 (0.71–0.96)
*Tubewell Ownership Type*		
tubewell located within *bari*	REF	
tubewell located in another *bari*	1.19 (1.03–1.37)	1.25 (1.07–1.46)
community tubewell	0.92 (0.72–1.18)	1.06 (0.81–1.39)
*Wealth Index*		
1 (poorest)	REF	
2	0.94 (0.80–1.11)	0.95 (0.80–1.12)
3	0.83 (0.71–0.98)	0.85 (0.73–1.00)
4	0.92 (0.78–1.08)	0.94 (0.80–1.11)
5 (richest)	0.88 (0.74–1.05)	0.91 (0.77–1.09)
*Mother’s Education*		
median years	0.99 (0.98–1.01)	1.00 (0.98–1.01)
*Seasonality*		
dry		
hot/summer	1.17 (1.04–1.32)	1.17 (1.03–1.31)
rainy	0.68 (0.59–0.78)	0.68 (0.59–0.78)
*Access to Institutions*		
NGO membership	1.10 (0.99–1.22)	
awareness of Union Information Center	0.92 (0.81–1.04)	
visited Union Information Center	0.86 (0.72–1.04)	
*Latrine Use*		
improved latrine (not shared)	REF	
improved latrine (shared)	1.03 (0.91–1.16)	
unimproved latrine	1.05 (0.90–1.21)	
*Flood Control*		
household outside embankment	0.83 (0.75–0.92)	
*Children under Five*		
number of children	1.43 (1.30–1.56)	1.43 (1.30–1.57)
*Imputed Variables*		
village level		0.34 (0.16–0.70)
*Bari* level		0.89 (0.78–1.03)

a Univariate (Crude) and multivariate (Adjusted) model results investigating the relationship between variables and relative risk of diarrheal diseases from a log-binomial GEE using an exchangeable correlation matrix and sandwich estimators to account for repeated observations. The multivariate model included tubewell use and the four confounders based on the DAG in Supplemental Figure 1. The adjusted model included 85419 observations (missing = 950). Data on variables not adjusted in the main model (Access to institutions, latrine use, and flood control) was available for 81937 observations (missing = 3532). Results of sensitivity analysis are available in .

Among different seasons, deep tubewell use was protective against diarrheal diseases during the dry (RR = 0.71, 95% CI: 0.52–0.97), and summer (RR = 0.82, 95% CI: 0.67–0.99) seasons but not the rainy season (Figure 3B). Among households outside the flood control embankment, using a deep tubewell resulted in the risk of diarrhea in children under five being 0.77 times (95% CI: 0.68–0.92) compared to shallow tubewell users. However, deep tubewell use, compared to shallow tubewell use, was not significantly associated with diarrhea in under-five children in households located within the embankment. Compared to shallow tubewell use, the association between deep tubewell use and child diarrhea risk was significantly protective among households using an unimproved latrine (RR = 0.62, 95% CI: 0.43–0.89), and among households using an improved latrine but sharing with other households (RR = 0.78, 95% CI: 0.60–0.99). The relationship between deep tubewell use compared to shallow tubewell use was protective and significant among households that did not visit a Union Information Center (RR = 0.80, 95% CI: 0.68- 0.94), but not among those that made a visit to these centers. Conversely, there was a significant protective relationship between deep tubewell use and diarrheal disease prevalence among households that were members of an NGO (RR = 0.74, 95% CI: 0.58–0.93), but not among those that were not.

## 4 Discussion

Our primary finding suggests that deep tubewell use compared to shallow tubewell use is associated with lower diarrheal disease prevalence among children under five years of age in our study area in rural Bangladesh. The direction of the association between deep tubewell use and diarrheal disease is consistent with the previous studies in Matlab^27,28^ and suggests that deep tubewell use may provide dual benefits of lower arsenic and diarrheal risk as compared to households that use shallow tubewells for drinking-water.

Importantly, our study also addresses the important limitations of previous studies. Escamilla et al. (2011)^28^ focused on only six villages in an area with high deep tubewell density and potentially missed variations in diarrheal disease risk in areas with limited deep tubewell access. Wu et al. (2011)^13^ and Winston et al. (2013)^27^ considered a larger study area comprising of 142 villages but assigned a proxy measure for tubewell access based on distance to tubewell and assumed that households used the same source over a period of multiple years. These assumptions can potentially fail to accurately measure household tubewell use. As demonstrated in our community surveillance, approximately one out of every 10 households reported using both shallow and deep tubewells at different time periods, as households may periodically switch to another source due to disruptions in the water supply or convenience during different seasons. Also, households may not always obtain drinking-water from their nearest tubewell, and instead prefer a source based on preferences such as perceived safety, convenience, and drinking-water taste, odor and color, among others.^30–32^ Hence, tubewell use may be more dynamic and time-varying than previously assumed. Finally, while distance to a water source is an important predictor of both diarrhea^42^ and the choice of drinking-water source,^5^ tubewell ownership is another important contextual factor.^33^ Although tubewell ownership is likely to be partially correlated to distance, it also encompasses other dimensions that may influence tubewell use. For example, some households may prefer to use a community deep tubewell instead of a deep tubewell in another nearby household compound, due to the fear of having to pay for a part of the owner’s tubewell repairs.^33^ By measuring dynamic tubewell use and considering factors such as tubewell ownership and seasonality across all of Matlab, our study provides a more representative and realistic association between deep tubewell use and diarrheal diseases among children under five in Matlab.

We also find that there are specific contextual factors that may further influence the association between deep tubewell use and diarrheal disease prevalence in children under five years of age in Matlab. Based on our stratum-specific estimates, we found that compared to shallow tubewell use, deep tubewell use is most protective when households are located outside the flood control embankment, use a tubewell during the dry season, or use an unimproved latrine. It has been found that tubewells are more prone to source contamination in flood-prone areas,^8^ and although deep tubewells have higher source contamination during monsoons, it is still lower than shallow tubewells due to higher source protection from greater aquifer depth. Similarly, compared to shallow tubewells, deep tubewells have very low source contamination during the dry season, but not during the rainy season.^14^ It has also been shown that shallow tubewells are at high risk of fecal contamination, especially in the presence of unsanitary latrines where fecal contamination may occur due to lateral transport.^11^ Hence, our results suggest that using a deep tubewell for drinking-water instead of shallow tubewells is most protective in contexts where there may be a higher risk of microbial contamination at the source among shallow tubewells. Notably, we also found that there was no significant difference in the socioeconomic status (SES) of households that used deep tubewells as compared to shallow tubewells and that SES may not be an important factor associated with tubewell type and diarrheal disease risk, in the context of Matlab. However, this may not be the case in other contexts of rural Bangladesh, where households that use deep tubewells, especially in their own compounds, may have lower diarrheal disease risk because they may be wealthier and more likely to afford the associated higher installation costs associated with deep tubewells.

Finally, we do not find support for the hypothesis that deep tubewell use may exacerbate diarrheal disease risk especially among under-five children in households that may procure drinking-water from distant deep tubewell sources. Previous evidence using microbial quality testing showed that despite having comparable water quality at source, households that used deep tubewells had comparatively higher microbial contamination at POU as compared to households that used shallow tubewells.^24^ Additionally, results of a large trial in rural Bangladesh found that households that switched to deep community wells had a higher likelihood of fecal contamination in their household drinking-water storage containers and that the likelihood of contamination was higher with each additional minute to reach the tubewell source.^23^ Hence, based on those previous findings, we would have expected that households using more distant community deep tubewells would have higher diarrheal disease risk compared to households using private shallow or deep tubewells, controlling for other confounders. However, we found that using community deep tubewells or those from another *bari*, compared to private shallow or deep tubewells, was not associated with higher risk of diarrheal diseases in under-five children.

While we did not measure microbial contamination at source or at POU during our community surveillance, there are multiple potential explanations for this null result. First, while microbial contamination at POU is an important risk factor for diarrheal disease morbidity and mortality, it may constitute only a small proportion of attributable risk, with higher risk attribution to other nondrinking water activities, and sanitation practices.^44,62^ Second, specifically in the context of rural Bangladesh where groundwater use for drinking-water consumption is very high, and households may have multiple drinking-water options within walking-distance, the relationship between higher POU microbial contamination due to farther walking distance may not have a large impact on diarrheal disease risk as compared to the differences in at-source microbial contamination risk between deep and shallow tubewells. In fact, we found that while comparing across different tubewell ownership strata, deep tubewell use, compared to shallow tubewell use, is notably associated with lower diarrheal disease prevalence when households must walk to a tubewell outside their *bari*. This suggests that despite farther distance and potentially higher POU microbial contamination, the general benefits of protection against at-source contamination for deep tubewells compared to shallow tubewells may play a larger role in reducing overall diarrheal disease prevalence.

However, we also note that compared to other areas of rural Bangladesh, the average household proximity to deep tubewells may be much higher in parts of Matlab. More than 40% of households in our community surveillance data used a deep tubewell within their *bari*, and a previous study indicated that on average, household members walked around 63 m to their preferred deep tubewell for procuring drinking-water.^24^ Hence, it is possible that the distance between households and deep tubewells may not be large enough to detect a measurable negative health effect of deep tubewell use on diarrheal diseases. In contrast, Buchmann et al. (2019),^29^ who found a negative association between deep tubewell use and mortality, conducted their study in the Barishal division, where more than 50% of households are estimated to not have drinking-water on their premises, and it is likely that households have to walk much further to access water from a deep tubewell, as compared to Matlab. It must be noted, however, that the authors only found a measurable increase in infant mortality risk among households that were more than 400 m away from a nearby deep tubewell, suggesting that deep tubewell use may be associated with negative health consequences primarily in circumstances where deep tubewells are much farther away and where there may not be other alternative sources of clean drinking-water. Additionally, although Barishal, similar to Matlab, was one of the areas most affected by groundwater arsenic contamination, the latest Bangladesh Multiple Indicator Survey suggests that only 1% of households in the Barishal division may be exposed to high arsenic contamination, as compared to 30% households in the Chattogram division (the division encompassing Matlab). Hence in this setting of lower arsenic contamination, the negative consequences of using a far away deep tubewell may outweigh potential benefits of better water quality at the tubewell source.

Our study also has important broader significance and policy implications. Diarrheal disease burden has persisted in rural Bangladesh with a prevalence of 5–8% ^38,63^ and deep tubewells remain a key option in the arsenal of interventions to ensure the provision of both microbially safe and arsenic-free drinking-water to millions of rural Bangladeshis. However, deep tubewell allocations have been inequitable and often opportunistic or politically driven, leading to elite capture.^64^ There are cost considerations too, given the relatively high cost of installing deep tubewells compared to shallow tubewells. Our findings suggest that despite concerns, children in households that do not have a deep tubewell in their *bari* may not be at higher risk of diarrheal disease if they procured drinking-water from an outside deep tubewell. Additionally, because households using a shallow tubewell in another *bari* are at higher risk of diarrheal disease, they may be able to lower their risk by instead switching to a deep tubewell even if it is not within their *bari*. Even though only 11% of shallow tubewell households used a shallow tubewell outside their own *bari*, this represents a sizable proportion (10%) of the population that is using shallow tubewells for drinking-water.

Similarly, our findings on stratum-specific associations between deep tubewell use and under-five child diarrhea across different variables can be utilized for targeted policy recommendations about future deep tubewell installations. For example, because the association between deep tubewell use and diarrhea is protective among households outside the embankment, deep tubewell density could be expanded in those areas to maximize the reduction in diarrheal disease risk through safe drinking-water use. Similarly, if households with existing unimproved latrines cannot afford to build improved latrines, using a deep tubewell instead of a shallow tubewell may decrease the diarrheal disease risk associated with fecal contamination of shallow tubewells due to lateral fecal transport.

Even in other strata, such as households residing inside the embankment, deep tubewell use may still be protective with lower, but possible potential for harm. Our results do not suggest that for example, deep tubewells should not be installed in those areas. Future policy decisions will need to balance the monetary costs associated with deep tubewell installation and the health and economic costs associated with arsenic-related morbidity and diarrheal diseases. Alternative strategies such as long-term funding of free arsenic testing for households using shallow tubewells may be more cost-effective than installing new deep tubewells for arsenic mitigation in some settings.^65^ However, in contexts where the risk of both microbial and arsenic contamination in the shallow aquifer is high, health and economic benefits associated with lower cases of childhood diarrhea may further offset large monetary costs associated with installing deep tubewells. Hence, while our findings would apply in other areas of rural Bangladesh where groundwater arsenic contamination is prevalent along with risk of high microbial contamination in the shallow aquifer, any scale-up of deep tubewell installations or large-scale switching through information campaigns would need to weigh alternative policy strategies.

We addressed important limitations in our study through multiple procedures. First, baseline variables used in the data set were derived from the 2014 HSEC. It is possible that from 2014 to 2018, some household characteristics may have changed. We compared recorded tubewell type in the 2014 HSEC among matched households that reported using only either a deep or a shallow tubewell throughout our study period in the community surveillance data. Among the matched households, 81% of households reported using the same tubewell type. Hence, we are more confident that our baseline characteristics from HSEC are representative of household level characteristics in 2018. Additionally, because GEE models require no missing data, we imputed baseline characteristics for over 20% households that did not match to the 2014 HSEC. We assigned composite *bari*-level measures, followed by village-level measures where households could not be matched to a *bari*. To check for stability of our estimates, we compared model estimates with a model with complete case observations without imputation and found similar estimates (Excel Table S4). The association between deep tubewell use and diarrheal disease was the same, but the upper confidence interval in the complete case model crossed the null due to loss of precision. Additionally, household-level analysis of under-five childhood diarrhea risk and tubewell use may also obscure individual level characteristics of children under five within the same household that may potentially influence the relationship between tubewell use and diarrheal disease risk. While we could not run an individual-level analysis, many individual-level characteristics of children under five such as age or sex would not confound the relationship between tubewell use and diarrheal disease. Similarly, in Matlab, the average number of under-five children per household was 1.19, with very few households having more than one under-five child.

Given the variation in deep tubewell installations across *baris* and villages, it is possible that the relationship between deep tubewell use and diarrheal disease varies across the space. The association between household-level deep tubewell use and childhood diarrhea is also likely to be influenced by neighborhood factors at the *bari* and village level. For example, tubewell density, a neighborhood-level factor has been shown to be negatively associated with diarrheal disease.^66^ Because the main objective of this paper is to evaluate the aggregate real-world effectiveness of deep tubewells in preventing or exacerbating diarrheal diseases, we did not account for those factors. This study did not measure and differentiate between the multiple pathogens that cause diarrhea that have different peaks during different seasons in different spatial settings.^67^ In a study of *shigellosis* (i.e., bacterial dysentery) in Matlab, it was found that two species of the same causative agent *Shigella*, a bacterium frequently associated with blood in stools during a diarrheal episode, were differentially associated with flood-controlled areas.^48^ While one species was more related to hygiene and sanitation, the other was more related to the environment. Because these variables are also related to deep tubewell use, it is likely that deep tubewell use will be differentially associated with the risk of different causative agents. In another area of rural Bangladesh, it was found that among children presenting with moderate to severe diarrhea, deep tubewell use was associated with higher odds of *Shigella sonnei* infections, one species of shigella pathogens causing shigellosis.^68^

## 5 Conclusion

Based on the current use and distribution of deep tubewells and diarrheal diseases in Matlab, Bangladesh, we found that the risk of diarrheal diseases in children under five years of age may be lower in households using deep tubewells as compared to children in households using shallow tubewells. Although there is evidence that households that drink water from deep tubewells have a higher likelihood of microbial contamination in their stored drinking-water,^25^ those findings do not translate to higher diarrheal disease among children younger than five years of age. Factors such as tubewell ownership, seasonality, location in a flood control area, latrine use, and access to economic and knowledge institutions can all influence the relationship between deep tubewell use and diarrheal disease. However, despite the perceived desirability of private installation of deep tubewells, use of deep tubewells for obtaining drinking-water is limited among rural Bangladeshis. Because deep tubewells are expensive, policy makers need to ensure safe drinking-water provision by balancing the cost and benefits associated with deep tubewell use. Future policies should consider more contextual and targeted approaches based on maximizing deep tubewell benefits in the context where they are most protective against diarrheal diseases.

## Supplementary Material





## Notes

Supporting figure (DOCX)
Supporting tables (XLSX)
