# Supplementary material for: Deep Tubewell Use and Child Diarrhea in Rural Bangladesh: Results from a Prospective Community Surveillance Study
Source: Environ Health Perspect. 2026 Apr 22;134(3):312–23. doi: 10.1021/EHP.6c00045 (PMC13347651; doi:10.1021/EHP.6c00045)

**Covariate selection**

We selected our confounding variables for the models based on a directed acyclic graph (DAG) as shown in Supplementary Figure 1. The DAG shows the hypothesized causal pathways and relationships through which tubewell type can affect diarrheal disease prevalence based on theory. By controlling for mother’s education, wealth, tubewell ownership, and seasonality, we estimate the association between using a deep tubewell and diarrheal disease through the causal pathway.

**Supplemental Figure 1**. Directed Acyclic Graph of relationship between tubewell type (green) and diarrheal disease prevalence(blue). The dark edges show the possible pathways after adjusting for confounders (orange)


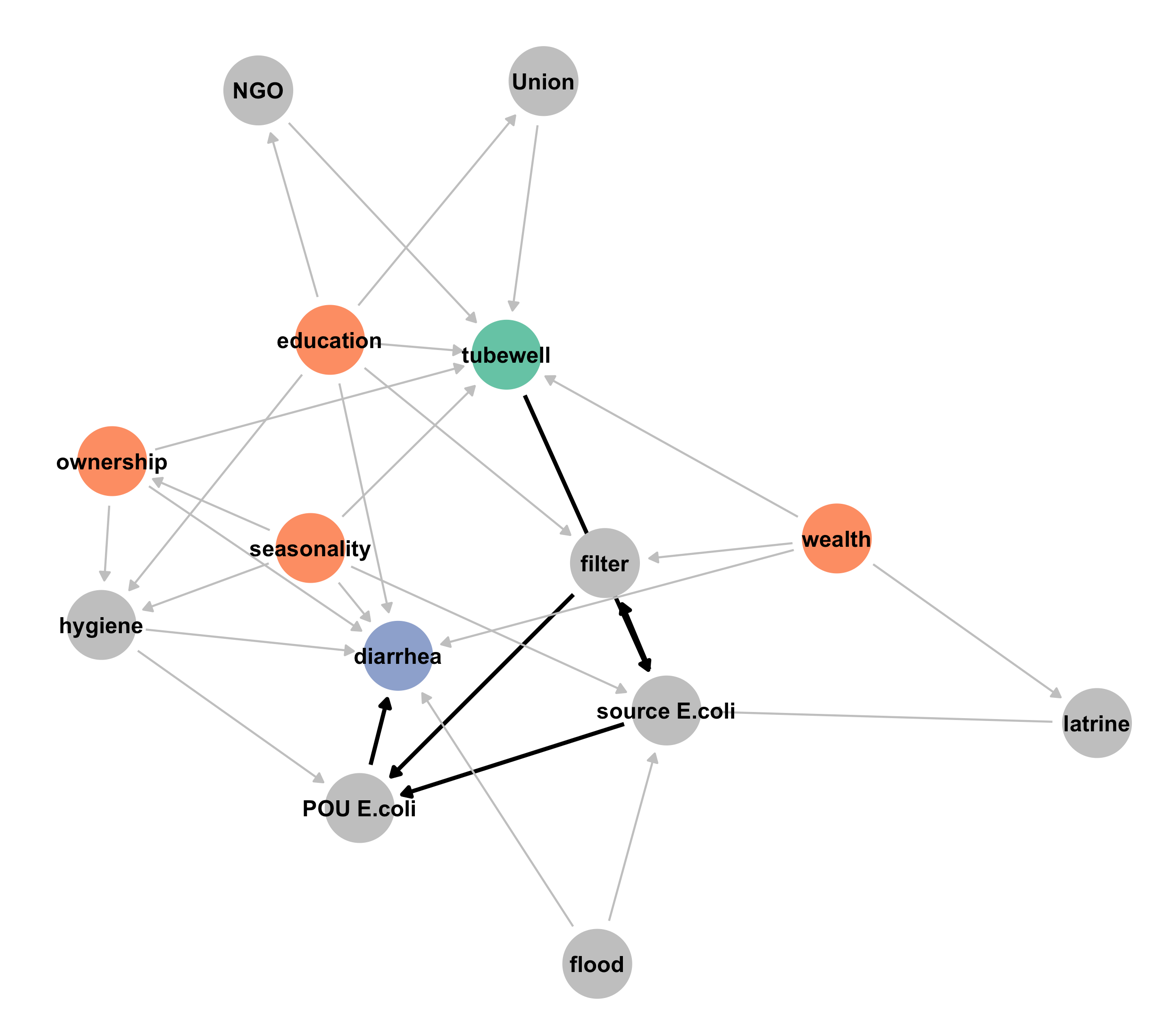

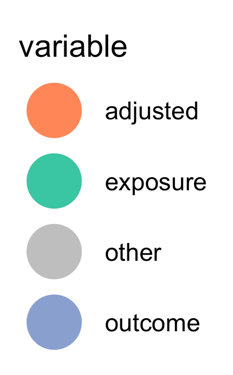

Supplement: Supplementary file 1 [file hp6c00045_si_001.docx]
